# Post-implantation shear stress assessment: an emerging tool for differentiation of bioresorbable scaffolds

**DOI:** 10.1007/s10554-018-1481-3

**Published:** 2018-11-13

**Authors:** Erhan Tenekecioglu, Ryo Torii, Yuki Katagiri, Ply Chichareon, Taku Asano, Yosuke Miyazaki, Kuniaki Takahashi, Rodrigo Modolo, Rasha Al-Lamee, Kadem Al-Lamee, Carlos Colet, Johan H. C. Reiber, Kerem Pekkan, Robert van Geuns, Christos V. Bourantas, Yoshinobu Onuma, Patrick W. Serruys

**Affiliations:** 1 Department of Interventional Cardiology, Erasmus University Medical Center, Thoraxcenter, Rotterdam, The Netherlands; 2 Department of Mechanical Engineering, University College London, London, UK; 3 Department of Cardiology, Academic Medical Center, University of Amsterdam, Amsterdam, The Netherlands; 4 Division of Cardiology, Department of Internal Medicine, Faculty of Medicine, Prince of Songkla University, Songkhla, Thailand; 5 International Centre for Circulatory Health, Imperial College London, London, UK; 6 Arterius, Leeds, UK; 7 Department of Cardiology, Universitair Ziekenhuis Brussel, Brussel, Belgium; 8 Department of Radiology, Leiden University Medical Center, Leiden, The Netherlands; 9 Department of Mechanical Engineering, Koc University, Istanbul, Turkey; 10 Department of Cardiology, University College of London Hospitals, London, UK; 11 Department of Cardiology, Barts Heart Centre, London, UK; 12 Imperial College, London, UK; 13 Dr.h.c. Melbourne School of Engineering, University of Melbourne, Melbourne (AUS), Westblaak 98, 3012KM, Rotterdam, The Netherlands

**Keywords:** Bioresorbable scaffolds, Shear stress, Computational fluid dynamics, Scaffold design

## Abstract

Optical coherence tomography based computational flow dynamic (CFD) modeling provides detailed information about the local flow behavior in stented/scaffolded vessel segments. Our aim is to investigate the in-vivo effect of strut thickness and strut protrusion on endothelial wall shear stress (ESS) distribution in ArterioSorb Absorbable Drug-Eluting Scaffold (ArterioSorb) and Absorb everolimus-eluting Bioresorbable Vascular Scaffold (Absorb) devices that struts with similar morphology (quadratic structure) but different thickness. In three animals, six coronary arteries were treated with ArterioSorb. At different six animals, six coronary arteries were treated with Absorb. Following three-dimensional(3D) reconstruction of the coronary arteries, Newtonian steady flow simulation was performed and the ESS were estimated. Mixed effects models were used to compare ESS distribution in the two devices. There were 4591 struts in the analyzed 477 cross-sections in Absorb (strut thickness = 157 µm) and 3105 struts in 429 cross-sections in ArterioSorb (strut thickness = 95 µm) for the protrusion analysis. In cross-section level analysis, there was significant difference between the scaffolds in the protrusion distances. The protrusion was higher in Absorb (97% of the strut thickness) than in ArterioSorb (88% of the strut thickness). ESS was significantly higher in ArterioSorb (1.52 ± 0.34 Pa) than in Absorb (0.73 ± 2.19 Pa) (p = 0.001). Low- and very-low ESS data were seen more often in Absorb than in ArterioSorb. ArterioSorb is associated with a more favorable ESS distribution compared to the Absorb. These differences should be attributed to different strut thickness/strut protrusion that has significant effect on shear stress distribution.

## Introduction

As a promising technology, bioresorbable scaffolds (BRS) introduced new terms such as *degradation, disappearance* and *recovery in vasomotricity*, into the interventionalists’ jargon. However, relatively high thrombosis events have been a serious setback in the development of this technology and jeopardized the *change in the paradigm* in percutaneous coronary revascularization [1, 2]. In the era of metallic DES, beside the implantation techniques, the stent design and strut thickness were deemed responsible for the adverse events [3, 4].

Stent/scaffold implantation creates a new endoluminal surface with near-wall blood flow interference that have major mechano-transduction impact [5]. Compared to pre-implantation, in instrumented vessel segments disturb the blood flow in relation to the scaffold design, particularly strut geometry and strut thickness [6, 7]. The blood rheology, local hemodynamic factors, prominently shear stress, play an important role in the vessel wall biology [5]. While laminar and relatively high shear stress is *athero-protective* and holds the platelets and endothelium *quiescent*, low and oscillatory shear stress upregulates inflammatory oxidative reactions, can induce thrombus formation, promote reactive neointima and subject the vessel wall to atherosclerotic changes [8, 9].

Optical coherence tomography (OCT) based computational fluid dynamics (CFD) model provides detail information on the local flow environment in stented/scaffolded vessel segments [10]. Our aim was to investigate in-vivo the effect of quadratic strut designs and strut protrusion on shear stress distribution in the vessel segments treated with BRS.

## Methods

### Study design and animal models

We analyzed data from Yucatan mini pigs implanted with Arteriosorb™ Absorbable Drug-Eluting Scaffold (ArterioSorb, Arterius Ltd., Leeds, UK) and Absorb everolimus-eluting Bioresorbable Vascular Scaffold (Absorb BVS, Abbott Vascular, Santa Clara, CA, USA). In three animals, six coronary arteries were treated with ArterioSorb and in other six animals, six coronary arteries were treated with Absorb. Study protocol was approved by the Institutional Animal Care and Use Committee of the testing facility (AccelLAB Inc., Boisbriand, Quebec, Canada) and were in compliance with the Canadian Council on Animal Care regulations. Animal husbandry, medication administration, and stent implantation were performed according to standards. The Testing Facility is accredited by the Association for Assessment and Accreditation of Laboratory Animal Care (AAALAC) and the Canadian Council on Animal Care (CCAC) [11].

### Scaffold design

**Fig. 1:**
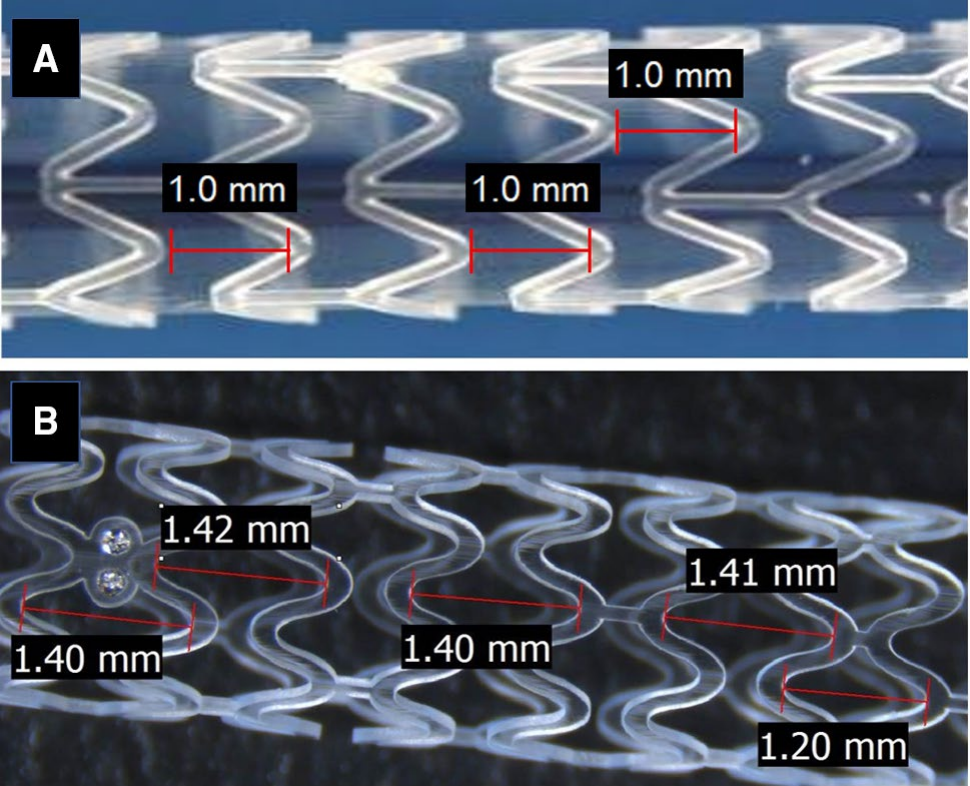
The design of the Absorb bioresorbable vascular scaffold (absorb) (**a**) and ArterioSorb bioresorbable scaffold (ArterioSorb) (**b**). In Absorb, the length of the cells are the same where as in ArterioSorb, the cells in the middle of the device are smaller than the cells in the proximal and distal of the scaffold

ArterioSorb is made up of poly l-lactic acid(PLLA), coated with a layer of poly d,l-lactic acid (PDLLA) eluting rapamycin (1.45 µg/mm^2^). ArterioSorb is manufactured using melt processing (extrusion) and die-drawing (solid-phase orientation) techniques with strut thicknesses of 95 µm and 120 µm. The strut width is 170 µm. ArterioSorb is composed of an 8-cell open-cell design with smaller cells at the center to improve the radial strength and cell connectors distributed in a spiral design (Fig. 1). The vessel coverage ratio in ArterioSorb is 29%. ArterioSorb has two pairs of radiopaque markers at distal and proximal edge of the scaffold.

Absorb is produced from PLLA, coated with a layer of a 1:1 mixture of an amorphous matrix of PDLLA and elutes everolimus (8.2 µg/mm^2^). Absorb is manufactured using extrusion and laser machining techniques, has 157 µm strut thickness and design of in-phase zig-zag hoops linked with bridges (Fig. 1). The strut width is 176 µm. The vessel coverage ratio of Absorb is 27%. Absorb has two pairs of radiopaque markers at distal and proximal edge of the scaffold.

### Scaffold implantation

To prevent any effect of swirling-flow due to vessel curvature, on the scaffolded segment ESS distribution, we didn’t include the cases with increased curvature. After pre-implant angiography was obtained, the target scaffold diameter was calculated from the reference vessel diameter which was 1.1 × (with a range of 1.05 × to 1.15 ×) the reference vessel diameter. Then the device was introduced into the selected artery by advancing the balloon catheter through the guide catheter and over the guidewire to the deployment site. The scaffolds were deployed according to the Interventionalist’s judgment using the product compliance charts and target vessel size as a guide, to achieve a targeted balloon-to-artery ratio of 1.1:1 with a range of 1.05:1 to 1.15:1. The balloon was not deployed below nominal pressure or higher than Rated Burst Pressure. The balloon was inflated slowly, at a 1 atm increment every 2 seconds until the scaffold is expanded. The final pressure should be maintained for at least 30 s. An angiogram of the balloon at full inflation was recorded and the maximal inflation pressure was noted. Post-dilatation was performed in all cases using a non-compliant balloon or by a semi-compliant balloon to ensure good apposition of the scaffold, and the target balloon to artery ratio met the requirements, for vessel diameters up to 3.20 mm at the discretion of the operator.

### Data acquisition

X-ray angiography was performed using Siemens Axiom cardiac angiography system (Siemens, Erlangen, Germany). For the post-implantation and post-dilatation angiographies, two projections with at least 25 degrees apart from each other were selected. When multiple balloon inflations were performed, the image with the highest pressure was used for analysis. Quantitative coronary angiography (QCA) measurements were obtained from these images using the QAngio® XA 7.3 system and Medis QCA-CMS 6.0 software (Medis, Leiden, The Netherlands).

OCT was performed before and after scaffold implantation in all treated coronary arteries, using a frequency-domain (FD) OCT system (Ilumien OCT Intravascular Imaging System; St. Jude Medical, St. Paul, MN, USA). When post-dilatation was performed, OCT was performed following each post-dilatation and at the end of the procedure to document OCT parameters at each successive stage. A non-occlusive flushing technique was used for blood clearance by injection of contrast media. After administration of nitrates (0.2 mg NTG intracoronary), the FD-OCT imaging catheter was advanced into the coronary artery in rapid exchange technique. The OCT catheter was advanced beyond the device, into the distal vessel, and pulled back to a point proximal to the device frequently to the ostium of the treated vessel. After FD-OCT catheter placement, blood was cleared by injection of iso-osmolar contrast. The FD-OCT pull-back was started as soon as the artery was cleared from blood and stopped when the imaging core reached the guiding catheter. Qualitative analysis of the captured images was then be performed (such as strut malapposition) off line. The acquired data were stored in DICOM format and transferred to a workstation for further analysis. The OCT analysis was performed using QCU-CMS software (version 4.69, Leiden University Medical Center, Leiden, The Netherlands) [12].

### Protrusion analysis by optical coherence tomography

For protrusion analysis, the protrusion distances were estimated semi-automatically using a special version of QCU-CMS software (version 4.69, Leiden University Medical Center, Leiden, The Netherlands) [12]. The protrusion analysis in OCT was performed in the scaffolded segment at every 200 µm longitudinal interval using a methodology presented previously [12]. Interobserver reproducibility analysis in protrusion in quadratic struts has been published previously [12].

### Coronary artery reconstruction

Coronary artery reconstruction was conducted implementing a validated methodology [13]. In X-ray angiographic and OCT images, anatomical landmarks (i.e. side branches) and the radiopaque markers were identified and used to define the scaffolded segment and proximal–distal native vessel segments. After matching, the OCT images portraying the scaffolded and the proximal–distal non-scaffolded native vessel segments were analyzed at a 100 micron (µm) interval in the scaffolded segment and 400 µm interval in the native vessel segments. The flow area was traced and defined in the native segments by the luminal border and in the scaffolded segments by the adluminal side of the struts and by the vessel luminal surface borders between the struts [14].

Two post-procedure end-diastolic angiographic images with at least 25º-angle difference showing the analyzed OCT frames (region of interest, ROI) with minimal foreshortening were selected. In these images, the luminal borders were delineated for the ROI and processed to extract the luminal centerline which was then used for the three-dimensional (3D) luminal centerline of the ROI [13]. The borders of flow area identified on OCT images were then mounted perpendicularly onto the luminal centerline and side-branches seen in both OCT and angiographic images were utilized to establish the absolute orientation of the OCT frames [13].

### Computational flow dynamics study

The reconstructed models were processed with CFD techniques. A finite volume mesh was generated and then blood flow simulation was performed. ESS was estimated by solving the 3D Navier–Stokes equations (ANSYS Fluent, Canonsburg, Pennsylvania) [15]. To assess the effect of scaffold designs on the local flow patterns, the mesh density around the struts and within the boundary layer of the flow between the struts was increased to have an average element edge equal to 1/5 of the strut thickness. Blood was assumed to be a homogeneous, Newtonian fluid with a viscosity of 0.0035 Pa.s and a density of 1050 kg/m^3^. A steady flow profile was simulated at the inflow of the 3D models. Blood flow for each reconstruction was estimated by measuring, in the 2 angiographic projections, the number of frames required for the contrast agent to pass from the inlet to the outlet of the reconstructed segment, the volume of the reconstructed segment and the cine frame rate [15]. The arterial wall was considered to be rigid and no-slip conditions were implemented at the scaffold surface. At the outlet of the model zero pressure conditions were imposed. ESS at vessel luminal surface was calculated as the product of blood viscosity and the gradient of blood velocity at the wall [16]. The ESS was measured in the native and the scaffolded segment around the circumference of the lumen per 5° interval (sector) and along the axial direction per 200 µm interval with the use of an in-house algorithm [16].

### Statistical analysis

Continuous variables were tested for normality distribution with Kolmogorov–Smirnov test and are presented as mean ± SD or median (interquartile range) as appropriate. Categorical variables are presented as counts and percentages. Continuous variables were compared by the Kruskal–Wallis test or Mann Whitney-U test. Categorical variables were compared by the Pearson Chi square test. Since the data have multi-level structure and unbalanced design, mixed effects model was used for statistical analysis. To compare the ESS values in different scaffold groups, the multi-level model was initially built with fixed effects on scaffold type, cross-sectional area and interaction of the scaffold type with cross-sectional area and random effects on animal ID, scaffold ID and cross-section ID. After comparing different models using maximum likelihood, best fitted model was selected. A p < 0.05 was considered statistically significant. Analyses were done using the statistical analysis program SPSS V.21, R V. 3.2.3 and the R package lme4 [17].

## Results

Three left anterior descending coronary artery (LAD) and 3 right coronary arteries (RCA) were implanted with an ArterioSorb and 1 LAD, 3 left circumflex (LCx) and 2 RCA with an Absorb. Scaffold dimensions, the flow velocities for each investigated vessel and procedural parameters are shown in Tables 1 and 2. In QCA, the dimensions of scaffolded segment and non-scaffolded segments are shown in Table 3. Mean lumen diameters and areas are comparable between the two scaffold groups.

**Table 1:** Scaffold dimensions and flow velocities for ArterioSorb and Absorb

Scaffold	Animal	Vessel	Scaffolded vessel segment	Scaffold size (mm)	Mean lumen diameter after post-dilatation (mm)	Flow velocities (m/s)
ArterioSorb-1	A	LAD	Distal	3.0 × 14	3.28	0.181
ArterioSorb-1	A	RCA	Mid	3.0 × 14	3.37	0.098
ArterioSorb-1	B	LAD	Mid	3.0 × 14	3.32	0.138
ArterioSorb-1	B	RCA	Proximal	3.0 × 14	3.00	0.135
ArterioSorb-1	C	LAD	Mid	3.0 × 14	3.16	0.155
ArterioSorb-1	C	RCA	Distal	3.0 × 14	3.20	0.147
Absorb BVS-1	E	RCA	Mid	3.0 × 18	3.32	0.104
Absorb BVS-2	D	LCx	Mid	3.0 × 18	3.06	0.103
Absorb BVS-3	F	LCx	Proximal	3.0 × 18	3.32	0.091
Absorb BVS-4	G	LAD	Mid	3.0 × 18	3.16	0.172
Absorb BVS-5	I	RCA	Mid	3.0 × 15	3.16	0.108
Absorb BVS-6	H	LCx	Mid	3.0 × 18	3.32	0.104

**Table 2:** Procedural details

Scaffold	Absorb (n = 6)	ArterioSorb (n = 6)	p
Implanted vessel			
LAD/LCx/RCA (n)	1/3/2	4/2/5	
Device			
Device nominal size (mm)	3.0 ± 0	3.0 ± 0	0.12
Device length (mm)	17.5 ± 1.22	14.00 ± 0	< 0.001
Maximum deployment pressure (atm)	7.0 ± 0	14.71 ± 2.94	
Pre-dilatation			
Pre-dilatation performed, n (%)	0 (0%)	0 (0%)	1.00
Post-dilatation			
Post dilatation performed, n (%)	6 (100%)	6 (100%)	1.00
Post dilatation balloon type			
Semi-compliant balloon, n (%)	0 (0%)	0 (0%)	
Non-compliant balloon, n (%)	6 (100%)	6 (100%)	1.00
Maximum post-dilatation balloon pressure (atm)	8.5 ± 2.7	16.2 ± 3.0	0.001
Maximum expected post-dilatation balloon size (mm)	3.37 ± 0.093	3.65 ± 0.59	0.08

**Table 3:** QCA variables in scaffold groups

	Absorb (157 µm) n = 6	ArterioSorb (95 µm) n = 6	p
Mean lumen diameter pre-implantation (scaffolded segment) (mm)	2.84 ± 0.15	2.83 ± 0.16	0.11
Mean lumen area pre-implantation (scaffolded segment) (mm^2^)	6.74 ± 0.45	6.58 ± 0.77	0.67
Mean lumen diameter post-implantation (scaffolded segment) (mm)	3.22 ± 0.11	3.03 ± 0.25	0.07
Mean lumen area post-implantation (scaffolded segment) (mm^2^)	7.49 ± 0.63	7.21 ± 1.58	0.69
Mean lumen diameter post-implantation (non-scaffolded segment) (mm)	3.19 ± 0.12	2.95 ± 0.19	0.045
Mean lumen area post-implantation (non-scaffolded segment) (mm^2^)	7.36 ± 0.85	6.83 ± 1.25	0.038

In OCT, in the device level analysis in-scaffold mean lumen area were comparable in the two scaffold groups. In the cross-section level analysis, in-scaffold mean lumen area was slightly higher in Absorb (7.62 ± 1.10 mm^2^) compared with the ArterioSorb (7.35 ± 0.86 mm^2^) (p = 0.052) (Table 4).

**Table 4:** OCT analyses results in scaffold groups

	Absorb	ArterioSorb	p
	(n = 6)	(n = 6)	
Device level			
In-scaffold mean lumen area (mm^2^)	7.77 ± 0.70	7.33 ± 0.69	0.074
Distal reference mean lumen area (mm^2^)	5.08 ± 1.31	6.19 ± 1.45	0.194
Proximal reference mean lumen area (mm^2^)	7.46 ± 2.63	7.81 ± 0.49	0.751
Mean scaffold area (mm^2^)	8.10 ± 0.61	7.76 ± 0.70	0.190

	(n = 478)	(n = 429)	
Cross-section level			
In-scaffold mean lumen area (mm^2^)	7.62 ± 1.10	7.35 ± 0.86	0.052
Distal reference mean lumen area (mm^2^)	5.07 ± 1.42	6.20 ± 1.41	0.465
Proximal reference mean lumen area (mm^2^)	6.85 ± 2.20	7.74 ± 0.93	0.843
Mean scaffold area (mm^2^)	8.00 ± 0.59	7.77 ± 0.87	< 0.0001

Data are expressed as n (%) and mean ± standard deviation

### Protrusion analysis

There were 4591 struts in 477 cross-sections from Absorb and 3105 struts in 429 cross-sections from ArterioSorb in the protrusion analysis. There were 128 and 25 malapposed struts in Absorb and ArterioSorb, respectively. The protrusion analyses were performed in device and cross-section levels (Table 5). In device level analysis, ArterioSorb struts protruded (84 ± 6 µm) in the vessel wall less than in Absorb (153 ± 18 µm) (p = 0.004). In cross-section level analysis, there was significant difference between the two scaffolds in strut protrusion (153 ± 137 µm for Absorb, 84 ± 12 µm for ArterioSorb; p < 0.0001), that can be attributed to difference in strut thicknesses. The protrusion was higher in Absorb (97% of the strut thickness) than in ArterioSorb (88% of the strut thickness). When the protrusion distance was adjusted according to the lumen diameters, the ratio of protrusion distance/mean lumen diameter was higher in Absorb BVS (0.052 ± 0.0038) compared with the ArterioSorb (0.028 ± 0.0045) (< 0.0001).

**Table 5:** Protrusion and ESS results

	Absorb	ArterioSorb	p
	(n = 6)	(n = 6)	
Device level			
Protrusion distance (µm)	153 ± 18	84 ± 6	0.004
Protrusion distance/mean lumen diameter	0.019 ± 0.002	0.012 ± 0.002	< 0.0001
In-scaffold mean ESS (Pa)	0.73 ± 0.25	1.51 ± 0.22	0.03
Proximal non-scaffolded segment mean ESS (Pa)	1.40 ± 0.06	1.08 ± 0.30	0.04
Distal non-scaffolded segment mean ESS (Pa)	1.31 ± 0.07	1.39 ± 0.25	0.26

	(n = 478)	(n = 429)	
Cross-section level			
Protrusion distance (µm)	153 ± 137	84 ± 12	< 0.0001
Protrusion distance/mean lumen diameter	0.052 ± 0.0038	0.028 ± 0.0045	< 0.0001
In-scaffold mean ESS (Pa)	0.73 ± 2.19	1.52 ± 0.34	0.001
Proximal non-scaffolded segment mean ESS (Pa)	n = 124 1.41 ± 0.36	n = 170 1.00 ± 0.47	0.355
Distal non-scaffolded segment mean ESS (Pa)	n = 147 1.29 ± 0.46	n = 260 1.39 ± 0.69	0.647

### Endothelial shear stress analysis

**Fig. 2:**
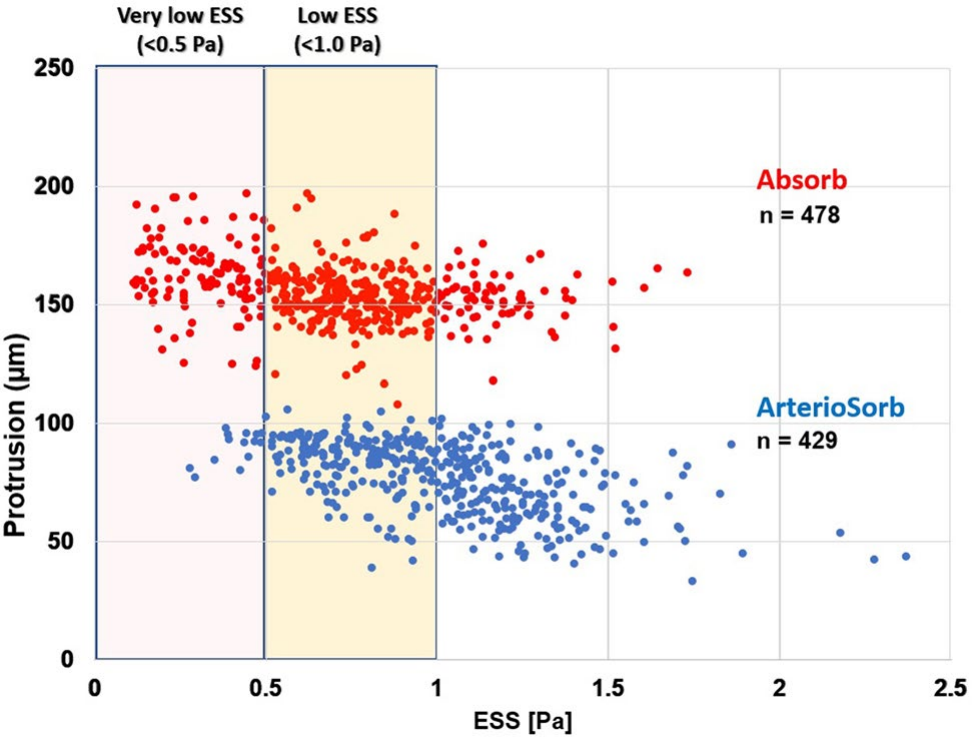
The low- and very-low ESS (median ESS per-cross-section) were recorded much more in Absorb than ArterioSorb

There were several layers of grouping within study data; nine animals (level 3) received scaffold implants in their coronary arteries, and similar types of the scaffolds were used in different vessels within the same animal. Each scaffolded segment had several cross-sections (level 2) and in each cross-section the ESS was quantified circumferentially in 5-degree sectorial subunits (level 1). ESS was significantly higher in ArterioSorb (1.52 ± 0.34 Pa) than in Absorb (0.73 ± 2.19 Pa) (p = 0.001) (Table 5). In both types of scaffolds, there were inverse correlations between strut protrusion and ESS. Low- (< 1.0 Pa) and very-low (< 0.5 Pa) ESS estimations were seen more often in Absorb implanted vessel segments as compare to the ArterioSorb (Fig. 2).

## Discussion

In this study, we investigated two different types of BRS with similar strut geometry but different strut thicknesses in porcine coronary artery models. The findings can be summarized as follows; (1) Strut protrusion was higher in Absorb (97% of the strut thickness) than in ArterioSorb (88% of the strut thickness). (2) The decreased protrusion resulted noted in ArterioSorb in higher ESS in these scaffolds compared to the Absorb. (3) Due to less strut protrusion in ArterioSorb, low and very low ESS were seen less in ArterioSorb than Absorb.

Stent/scaffold induced hemodynamic changes are one of the decisive determinants of PCI outcomes [13, 15, 18–20]. Several factors, such as vessel curvature in the treated segment, residual stenosis following device implantation, local deformation at the edges of the implanted device, tissue protrusion between the struts, scaffold design properties are factors responsible for the hemodynamic repercussion following stent/scaffold implantation. Among these factors, stent/scaffold design and strut apposition status are of utmost importance due to a relationship between new established luminal surface and local flow behaviors. Shear stress has been demonstrated to be inversely related with neointimal healing process following stent/scaffold implantation [21]. In this relationship, strut thickness and protrusion emerge as cornerstone factors for the flow behaviors post-PCI [22]. Besides the micro level changes around the struts, fluid shear stress at a macro level is influenced by the vessel luminal dimensions after stent/scaffold implantation.

**Fig. 3:**
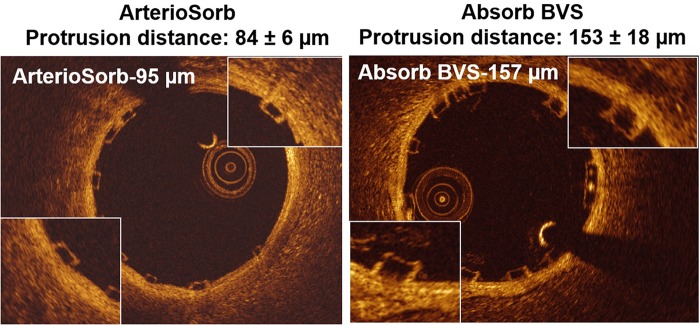
The protrusion in ArterioSorb was less than Absorb, due to the thinner struts of ArterioSorb

**Fig. 4:**
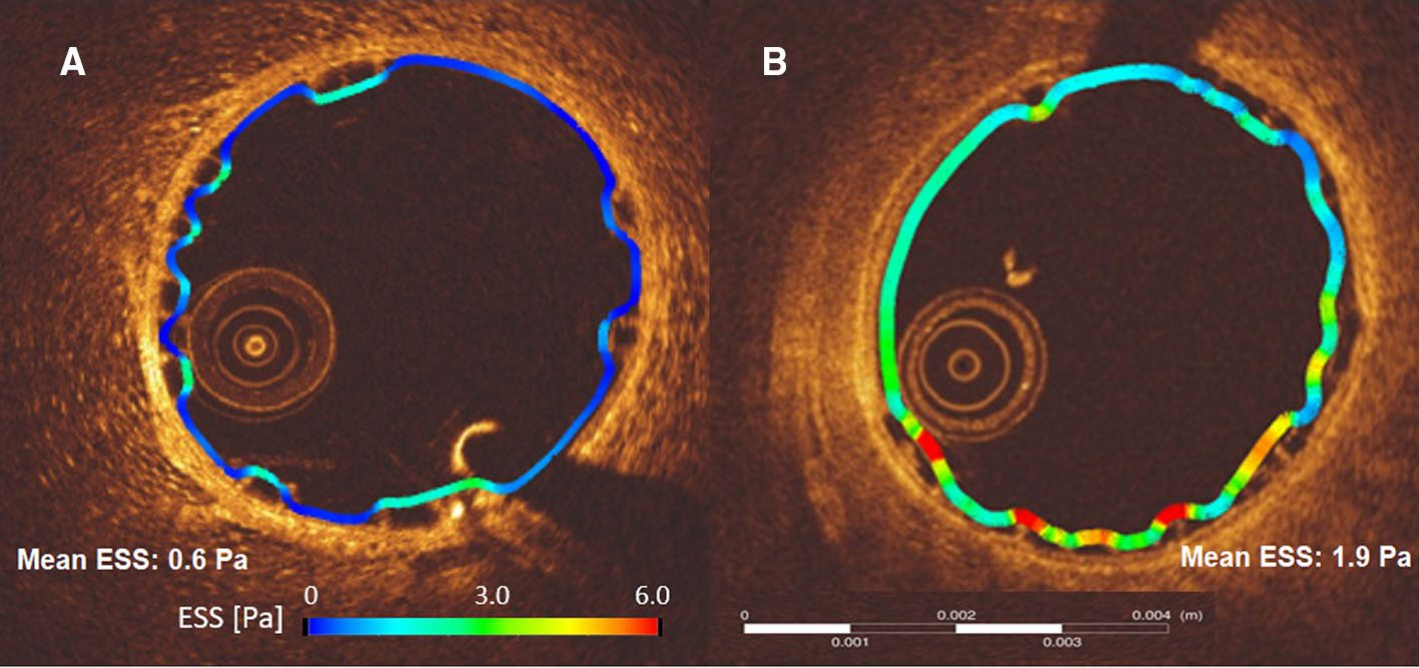
Due to thinner struts in ArterioSorb (**a**), the shear stress magnitudes were relatively higher than in Absorb (**b**). In the cross-sections from Absorb and ArterioSorb, mean ESS were 0.6 Pa and 1.9 Pa, respectively

In the present report, two types of scaffolds were implanted in straight vessels. Both scaffolds have square-shaped strut designs, however ArterioSorb has thinner strut thickness of 95 µm which obviously induces less flow separations compared to thicker struts of Absorb (Fig. 3) [6, 23]. Less flow separation will subject the newly set vessel surface to relatively higher endothelial wall shear stress (Fig. 4). In ArterioSorb, the final luminal area post-implantation was slightly smaller than the Absorb and there was an obvious difference in the strut protrusion between the scaffolds. After adjusting the protrusion distances according to the final luminal dimensions, ArterioSorb had lower ratio of protrusion distance/luminal diameter as compared to the Absorb.

**Fig. 5:**
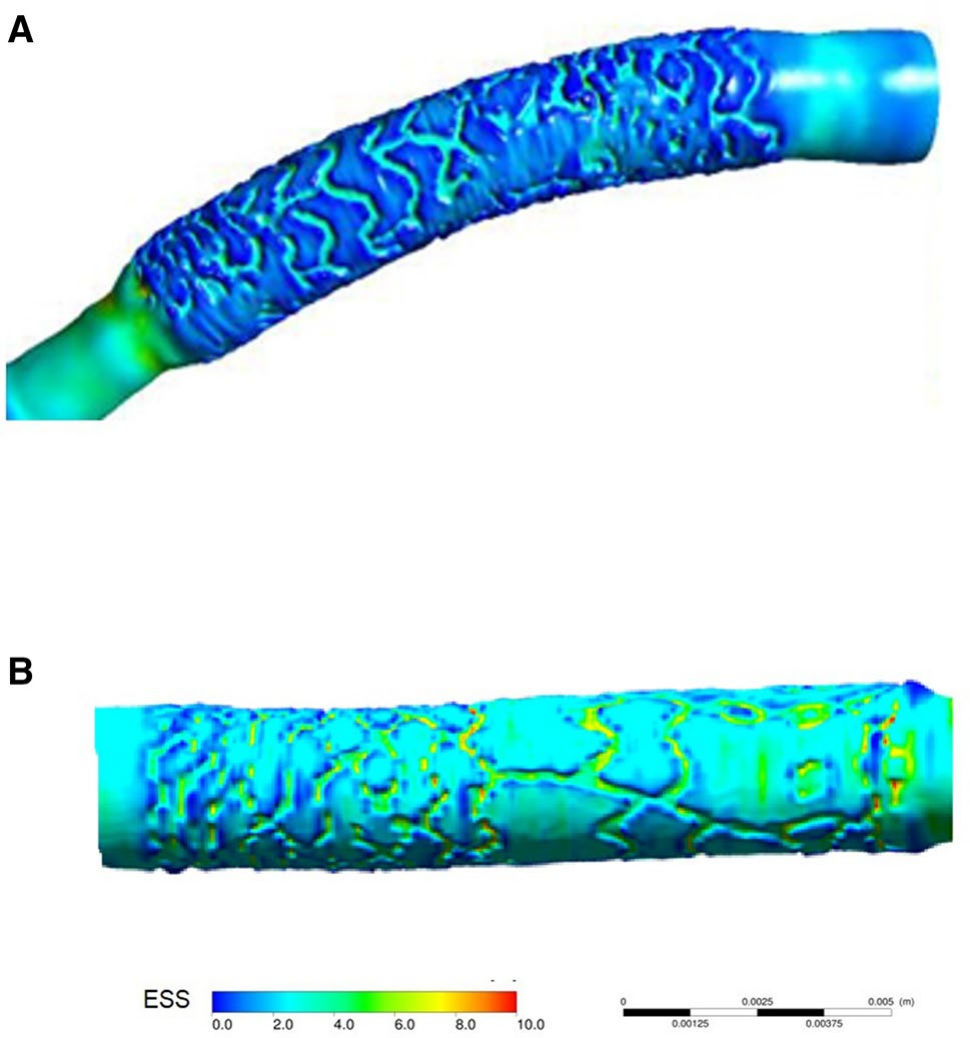
The inter-strut distance is 1.0 mm in Absorb (**a**) whereas 1.4 mm in ArterioSorb (**b**). The wider inter-strut distance provides more space to ArterioSorb to regain laminar flow and more physiological ESS over the wall boundary

The inter-strut distance has impact on the recovery of the laminar flow between the struts [24]. The inter-strut distance should be at least six times the strut thickness [24]. ArterioSorb has maximum inter-strut distance of 1.4 mm which is longer than in Absorb (1.0 mm) and provides a significant advantage for the recovery of laminar flow resulting in more physiological ESS between the struts (Fig. 5).

Bare metal stent (BMS) studies have emphasized that stent design influences clinical outcomes [3, 25]. Anti-proliferative drugs has changed the paradigm in drug-eluting metallic stents as the drug reduced the vessel wall proliferative reaction and prolonged vessel wall healing process. In BRS, the findings of the recently reported clinical studies raised concerns about the potential risk of scaffold thrombosis that have been at least partially attributed to BRS design [26]. The non-streamlined strut design has significant effects on local flow patterns even in highest coronary flow that cannot be omitted [26, 27]. To address this limitation, industry has focused on more “hemocompatible” scaffold designs with different strut thicknesses, alignment and geometry. The present study has shown that in vivo OCT based CFD modeling can be used to investigate the hemodynamic performance of different scaffolds. CFD may have a significant role in the optimization of the scaffold design in favor of coronary flow [28]. In addition to high-tech CFD studies, protrusion analysis on OCT appears to contribute to optimized implantation which is critical for satisfactory results at follow-up after PCI [29, 30].

### Limitations

A major limitation of the present study is the fact that scaffold implantation was performed in healthy coronary arteries. Therefore, it was not possible to investigate the implications of scaffold under-expansion or the effect of the underlying treated plaque on strut protrusion which is likely to determine the local hemodynamic features.

Secondly, one of the main limitation was the small sample size. Several criteria were implemented for filtering suitable cases. To prevent any effect of swirling-flow due to vessel curvature, on the scaffolded segment ESS distribution, we didn’t include the cases with curvature. Also, the cases without two angiographic projections at least with > 25-degree difference couldn’t be reconstructed. However, total strut and cross-section numbers provided well-fitted statistical models for getting reliable conclusions.

Thirdly, the treated vessels (LAD, LCx and RCA) were different and there was an uneven distribution for the vessels treated between the study groups. Moreover, vessel diameters, the deployment pressures and the balloon pressures during post-dilation were different which might induce potential differences in the flow between the treated segments.

It’s well known that side-branches can impact on the flow and shear stress distribution in the scaffolded segment [7]. One of the limitations of this study was that we did not include in the reconstructions of the side branches. However, there was no side branches bigger than 1.5 mm in the scaffolded segments in our data which could dissipate the concerns about that point.

## Conclusion

In vivo OCT based CFD modeling can be used to evaluate the implications of scaffold configuration on the local hemodynamic forces. The thinner strut of ArterioSorb has less effect on the ESS patterns compared to the thicker struts of the Absorb. Further research is required to examine the potential value of the in vivo CFD modeling in optimizing scaffold configuration and clinical outcomes.
